# Synthesis and characterization of modified κ-carrageenan for enhanced proton conductivity as polymer electrolyte membrane

**DOI:** 10.1371/journal.pone.0185313

**Published:** 2017-09-28

**Authors:** Joy Wei Yi Liew, Kee Shyuan Loh, Azizan Ahmad, Kean Long Lim, Wan Ramli Wan Daud

**Affiliations:** 1 Fuel Cell Institute, Universiti Kebangsaan Malaysia, Bangi, Selangor, Malaysia; 2 School of Chemical Sciences and Food Technology, Faculty of Science and Technology, Universiti Kebangsaan Malaysia, Bangi, Selangor, Malaysia; 3 Department of Chemical and Process Engineering, Faculty of Engineering and Built Environment, Universiti Kebangsaan Malaysia, Bangi, Selangor, Malaysia; University of Akron, UNITED STATES

## Abstract

Polymer electrolyte membranes based on the natural polymer κ-carrageenan were modified and characterized for application in electrochemical devices. In general, pure κ-carrageenan membranes show a low ionic conductivity. New membranes were developed by chemically modifying κ-carrageenan via phosphorylation to produce O-methylene phosphonic κ-carrageenan (OMPC), which showed enhanced membrane conductivity. The membranes were prepared by a solution casting method. The chemical structure of OMPC samples were characterized using Fourier transform infrared spectroscopy (FTIR), ^1^H nuclear magnetic resonance (^1^H NMR) spectroscopy and ^31^P nuclear magnetic resonance (^31^P NMR) spectroscopy. The conductivity properties of the membranes were investigated by electrochemical impedance spectroscopy (EIS). The characterization demonstrated that the membranes had been successfully produced. The ionic conductivity of κ-carrageenan and OMPC were 2.79 × 10^−6^ S cm^-1^ and 1.54 × 10^−5^ S cm^-1^, respectively. The hydrated membranes showed a two orders of magnitude higher ionic conductivity than the dried membranes.

## Introduction

An important component in electrochemical devices is the polymer electrolyte membrane (PEM), which is an ion-conducting membrane with moderate-to-high ionic conductivity (≤ 10^−4^ S cm^-1^) at room temperature [1]. Since the discovery of the first ion-conducting polymer, namely, poly(ethylene oxide) (PEO) complexed/dissolved with alkali metal salts, different types of polymers have been investigated for potential use as PEMs. In recent years, research has extended to natural polymers due to their low- cost, availability and biocompatibility compared to synthetic polymers. Polysaccharides are the most abundant natural polymer in the environment. The most widely studied polysaccharides for PEM applications are starch, cellulose and chitosan [2–4]. The use of chitosan as a polymer electrolyte is noteworthy. Chitosan polymer electrolytes in salt complexes, chemically modified chitosan and chitosan blended with other polymers have been reported in the literature for application in electrochemical devices [5–7]. However, carrageenans have rarely been used as polymer electrolytes. The present works uses κ-carrageenans as a polymer electrolyte due to its chemical structure, which is similar to cellulose or chitosan.

Carrageenans which have molecular masses ranging from 400–600 kDa [8], are mainly used in the food industry as gelling, thickening and stabilizing agents and and are also used in cosmetics, paints, pharmaceuticals and the biomedical industry [9, 10].

**Fig 1:**
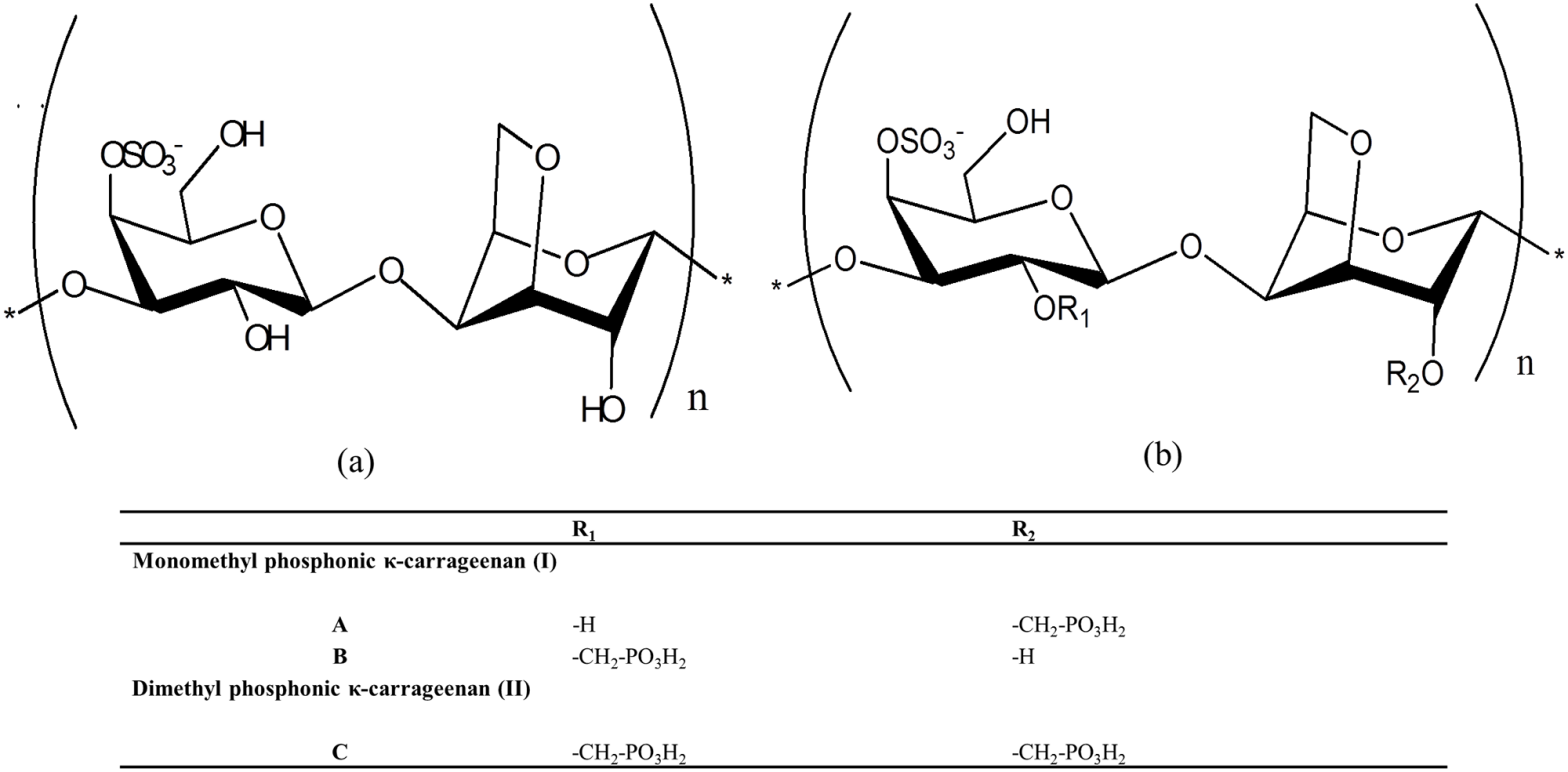
Molecular structure of (a) κ-carrageenan and (b) O-methylene phosphonic κ-carrageenan.

Carrageenans are natural water-soluble linear sulphated polysaccharides extracted from red seaweed [8], The predominant carrageenan extracted from Eucheuma cottonii is κ-carrageenan (Fig 1a), which consists of alternating repeating units of 4-linked 3,6-anhydro-α-D-galactose and 3-linked β-D-galactose with one sulphate per disaccharide unit. The considerable amount of sulphonic groups in the structure of κ-carrageenan allows films to form through the self-aggregation of its helical structures [11, 12]. The films possess good transparency, tensile strength and gelling ability [13, 14]. Other advantages, such as low energy consumption, low-cost synthesis, carbon neutrality and harmless disposal have also enhanced the potential of carrageenan as a polymer electrolyte. κ-carrageenan has high proton conductivity, in which the protons migrate through its hydrogen-bonded network, because of its hydrophilic nature, which promotes water absorption. [12]. The structure of κ-carrageenan consists of hydroxyl groups, which enables the formation of coordinate bonds with cations or protons [15]. Proton conductivity is caused by the transport of protons along the polymer structure of κ-carrageenan. Normally, pure polysaccharides have relatively low proton conductivity.

There are two ways to increase the ionic conductivity of a polymer: improving the polymer chain mobility or increasing the carrier concentration [16]. Here, a chemical modification of κ-carrageenan is proposed to modulate its physicochemical properties [17]. An ionogenic functional group is introduced into κ-carrageenan to obtain a highly negatively charged membrane and increase its ionic conductivity [6]. Modified κ-carrageenan has been previously explored as a polymer membrane, and the addition of a new functional group has shown positive results in improving the proton conductivity [18–20].

Wan et al. demonstrated that the phosphorylation of chitosan is able to increase its ionic conductivity by one order of magnitude [7], providing a good basis to explore the potential of phosphorylated κ-carrageenan as a polymer electrolyte, which to the best of our knowledge, has not yet been explored. Herein, methylene phosphonic acid was introduced into κ-carrageenan as a phosphoryl functional group to produce phosphorylated κ-carrageenan.

The ionic conductivity of the O-methylene phosphonic derivative of κ-carrageenan (OMPC) was enhanced as the modification of κ-carrageenan increased the number of oxygen atoms, which acts as electron donor groups to form coordination bonds with protons. The preparation and characterization of OMPC powder are discussed herein the study. The predicted structure is shown in Fig 1b. The potential of modified κ-carrageenan as a PEM was investigated.

## Materials and methods

### Materials

Commercial grade κ-carrageenan (TA150) was obtained from Tacara Sdn. Bhd., Tawau, Malaysia. Both phosphorous acid and formaldehyde solution were purchased from Sigma-Aldrich. Acetic acid was supplied by Merck, and acetone was supplied by Systerm. All materials were of analytical grade and were used without further purification. Deionized water was used throughout the experiment.

### Synthesis of O-methylene phosphonic κ-carrageenan

O-methylene phosphonic κ-carrageenan (OMPC) was prepared with some modifications according to a previously reported phosphorylation method [21]. κ-carrageenan solution was prepared by dissolving 10 g of κ-carrageenan in 500 mL 1% (v/v) glacial acetic acid. Then, 5 g of phosphorous acid was dissolved in 50 mL deionized water. The κ-carrageenan solution was heated to 70°C under reflux. Both the phosphorous acid solution and 5 mL of formaldehyde were added simultaneously to the κ-carrageenan solution. The temperature of the solution was maintained for 8 h. The pale yellow solution was then cooled to room temperature, followed by the addition of acetone into the solution to precipitate the OMPC powder. The resulting precipitate was filtered by a vacuum pump and washed with acetone in a Soxhlet apparatus for 24 h to remove the unreacted phosphorous acid. Finally, the precipitate was dried in a desiccator.

### Preparation of the membranes

The membranes were prepared by a solution casting method [19]. κ-carrageenan and OMPC solutions were prepared by dissolving the compounds in 1% (v/v) aqueous acetic acid solution, respectively [19]. The mixtures were continuously stirred with a magnetic stirrer for 24 h at room temperature. Then, the solutions were cast in Teflon dishes and placed in a fume hood for film formations. Films were obtained after drying at room temperature for 72 h and were then placed in a desiccator for further drying.

### Sample characterization

FTIR analysis was conducted using a Nicolet 6700 spectrophotometer in the range of 4000–650 cm^-1^ to determine the functional groups of the products. The spectrophotometer was equipped with an Attenuated Total Reflection accessory. ^1^H-NMR and ^31^P-NMR were performed using a Bruker Avance 111 600 MHz NMR spectrometer by dissolving the samples in deuterium oxide (D_2_O) to confirm the structure of modified κ-carrageenan.

The degree of substitution (DS) of κ-carrageenan and OMPC was determined by the saponification method [3]. For this, 0.1 g of κ-carrageenan or OMPC was dissolved in 50 mL of 0.3 M NaOH solution, and the mixture was continuously stirred for 24 h. Then, phenolphthalein indicator was added and the unreacted NaOH was back-titrated with 0.3 M HCl solution. κ-carrageenan served as a blank because the sulphonic group also acts as the charge transferring functional group. The titration was repeated three times, and the average value was used to calculate the DS.

For OMPC, the methylene phosphonic (MP) content was calculated as:

$$\text{MP}\left(\%\right)=\;\left[\left({\text{V}}_{\text{n}}-{\;\text{V}}_{\text{o}}\right){\text{C}}_{\text{HCl}\&\text{NaOH}}{\text{M}}_{\text{MP}}\right]/10\text{w}$$ (1)

where V_n_ is the volume of 0.3 M HCl consumed during the back-titration of OMPC, V_o_ is the volume of 0.3 M HCl used to titrate the blank, w is the weight of the sample in gram. M_MP_ is the molecular weight of the grafted methylene phosphonic residue, and C_HCl&NaOH_ is the concentration of the NaOH and HCl solution. The DS of OMPC was calculated using the formula:

$$\text{DS}=\frac{385.36\;\times \text{MP}}{\left[100{\text{M}}_{\text{Mp}}-\text{MP}\left({\text{M}}_{\text{MP}}-1\right)\right]}$$ (2)

where 385.36 is the molecular weight of the κ-carrageenan monomer.

A Bruker D8 Quest SC-XRD X-ray diffractometer was used to analyse the degree of crystallinity of the samples. The diffraction angle 2θ ranged from 3° to 60°.

A Perkin Elmer STA6000 TGA was used to test the thermal behaviour of the polymer to understand the thermal stability of the polymer. Both the κ-carrageenan and modified κ-carrageenan (15–20 mg) sample were tested under N_2_ gas atmosphere at a flow rate of 20 mL/min and a heating rate of 10°C/min, from ambient temperature to 600°C. A Perkin Elmer STA6000 differential scanning calorimeter was used to investigate the thermal transitions of the samples. The test was performed under N_2_ gas atmosphere from ambient temperature to 200°C at a heating rate of 10°C/min.

The morphology and elemental analyses of the samples were determined using a variable-pressure scanning electron microscope (VPSEM) coupled with energy-dispersive X-ray (EDX) spectroscopy using ZEISS EVO MA(UK) and EDAX APPOLO X(USA) at 10 kV with a magnification of 5000×.

Electron impedance spectroscopy was performed at room temperature in the frequency range of 1 Hz to 1 MHz with a 10 mV amplitude using an Autolab potentiostat AUT128N Frequency Response Analyzer. The ionic conductivity of the samples was measured using a two-electrode setup at a room temperature and humidity of approximately 52%. The graph of the negative imaginary part of the impedance (Z”) against the real part of the impedance (Z’) was plotted. The bulk resistance, R_b_, was obtained from equivalent circuit analysis using the Nova software. The ionic conductivity, σ, was calculated using the equation:

$$\sigma =\frac{\text{t}}{{\text{R}}_{\text{b}}\text{A}}$$ (3)

where t is the film thickness in cm measured using a hand-held micrometer, and A is the active area of the electrode in cm^2^. The thickness of the membranes is in the range of 50 μm to 200 μm. The surface contact area is 2.011 cm^2^, and the film was sandwiched between two identical stainless steel electrodes under spring pressure. To analysis the hydrated membranes, the membranes were immersed in deionized water and then quickly placed between the electrodes in the measurement cell. The water content was assumed to remain constant during the measurement.

## Results and discussion

### Characterization of κ-carrageenan and OMPC

**Fig 2:**
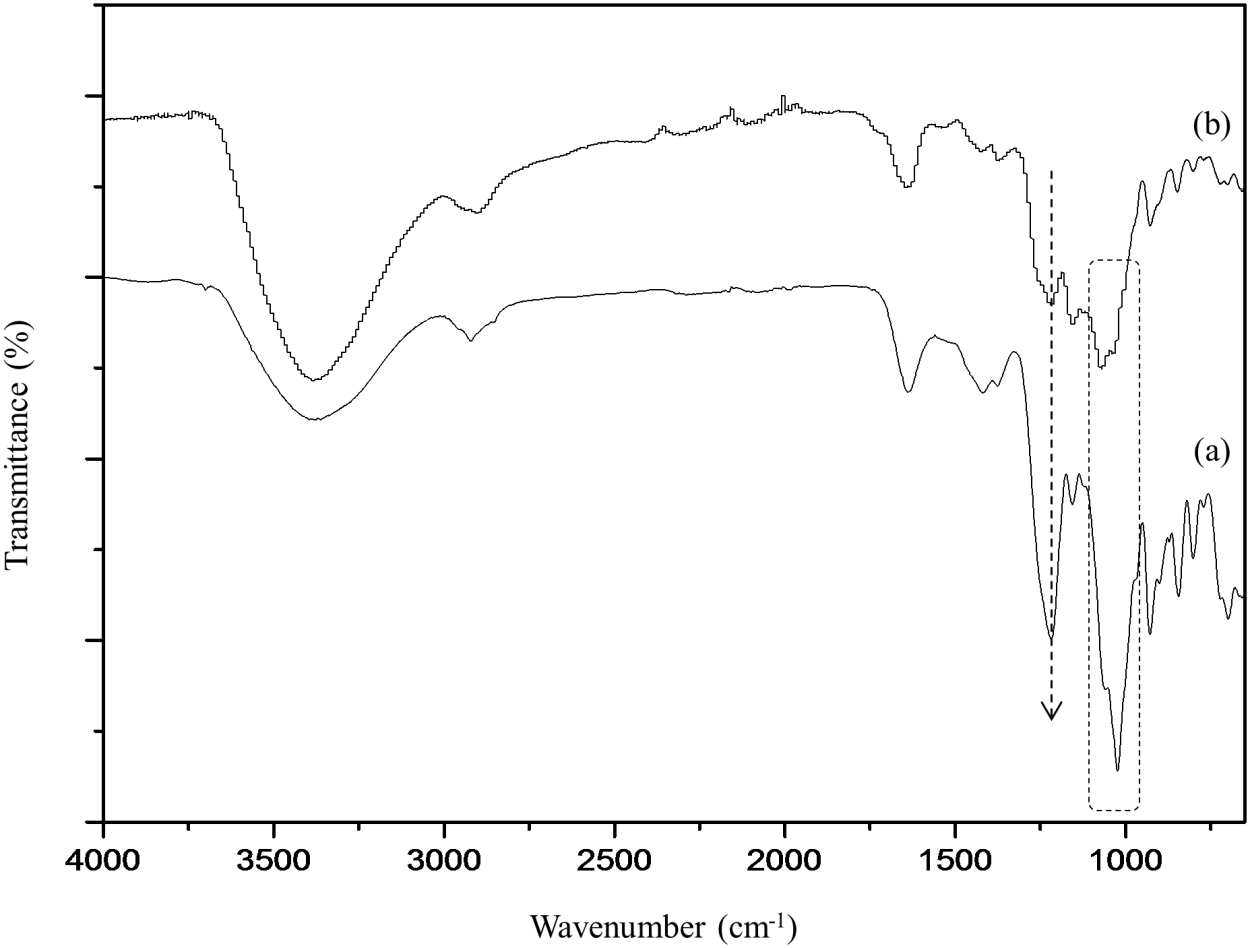
FTIR spectra of (a) κ-carrageenan and (b) OMPC.

The FTIR spectrum of κ-carrageenan (Fig 2a) shows a broad absorption peak at 3378 cm^-1^, indicating the OH stretching mode. The characteristic bands of κ-carrageenan occurred at 1219 cm^-1^ and 844 cm^-1^, representing the O = S = O stretching vibration mode and the –O-SO_3_ stretching vibration mode at the C-4 position of galactose, respectively [1, 19]. The bridge C-O stretching mode is displayed by the absorbance peak at 1156 cm^-1^ [19, 22]. The intense peak at 1024 cm^-1^ represents the C-O stretching mode. The 929 cm^-1^ absorption peak is characteristic of the C-O-C vibration mode of the 3,6-anhydro-D-galactose residue [22]. The signals at 1418 cm^-1^ and 1376 cm^-1^ are due to the C-O-H in-plane bending vibration mode and C-H bending vibration mode, respectively. In comparison with the FTIR spectrum of κ-carrageenan, the absorption bands at 1216 cm^-1^ and 1151 cm^-1^, which represent the O = S = O and C-O stretching modes, respectively, overlap and are shifted to slightly lower frequencies in the OMPC spectrum (Fig 2b). This change is due to the presence of P = O asymmetric stretching and –C-P- absorption [23, 24]. The new peak at 1061 cm^-1^, which represents the ν P-OH overlap with the C-O stretching mode at 1030 cm^-1^, results in a broader absorption peak [6, 25, 26]. The overlapping of the peaks and the presence of the new peak demonstrate the substitution of the methylene phosphonic group onto κ-carrageenan. This result is further confirmed by ^1^H NMR and ^31^P NMR spectroscopy.

**Fig 3:**
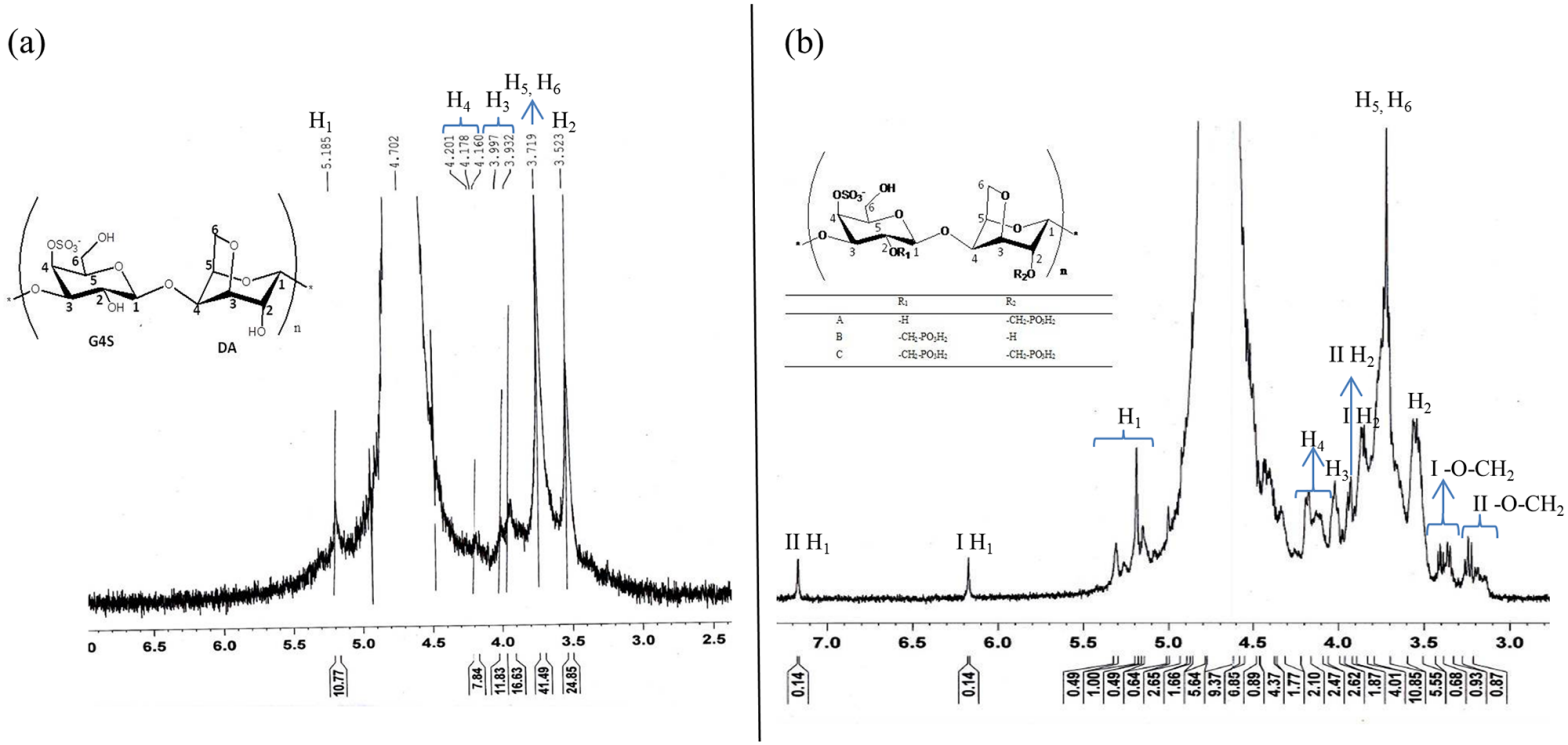
^1^H NMR spectra of (a) κ-carrageenan and (b) OMPC.

^1^H NMR spectroscopy provides insight into the structural properties of modified κ-carrageenan. We know that κ-carrageenan is a disaccharide polymer with alternating 3-linked β-D-galactopyranose (G-units) and 4-linked 3,6-anhydro-α-D-galactopyranose (DA-units) repeating units. The spectrum in Fig 3a shows the following chemical shifts: ^1^H NMR δ = 5.19 (H1), δ = 4.16–4.20 (H4), δ = 3.93–4.00 (H3), δ = 3.72 (H5, H6) and δ = 3.52 (H2), which are in agreement with the literature [17, 19, 27, 28]. The assignments and chemical shifts in the ^1^H NMR spectrum of OMPC (Fig 3b) confirmed the hypothesis that the introduction of methylene phosphonic groups in κ-carrageenan produces monomethylphosphonic κ-carrageenan (I) and dimethylphosphonic κ-carrageenan (II), as shown in Fig 1b. These two forms of κ-carrageenan (I and II) are distinguishable due to the presence of two new peaks at 3.35–3.42 ppm and 3.23–3.26 ppm, respectively, which are contributed by the methylene phosphonic group (-CH_2_PO_3_H_2_) [24]. The hydroxyl group in κ-carrageenan contains a lone pair of electrons that enables the methylene phosphonic group to replace the hydrogen atom. Thus, the ^1^H NMR spectrum shows two different chemical shifts for H1 at 6.17 and 7.17 ppm, and the downfield chemical shifts for H2 confirmed the introduction of -CH_2_PO_3_H_2_ at the C-2 position in the chemical structure of κ-carrageenan to produce OMPC [29, 30].

**Fig 4:**
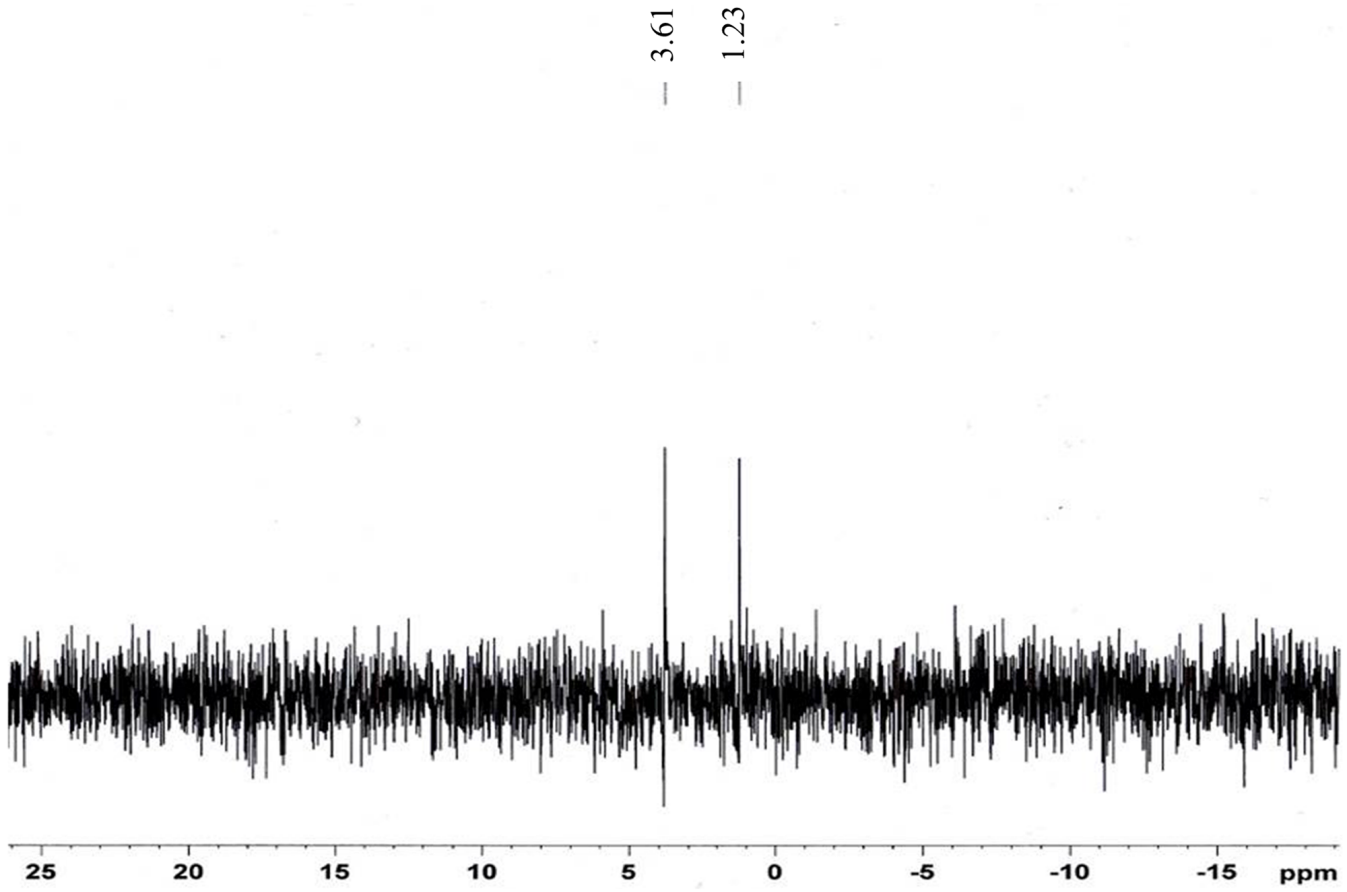
^31^P NMR spectrum of OMPC.

The replacement of H in H_3_PO_3_ by –CH_2_ causes a downfield shift in the spectrum [21]. The ^31^P NMR spectrum (Fig 4) shows two distinct peaks at 3.81 ppm and 1.23 ppm, further confirming the substitution of the methyl phosphonic group into κ-carrageenan to produce OMPC. The peak at 3.81 ppm is suggested to be –CH_2_PO_3_H_2_ attached to the polymer chain. The presence of the phosphate group was proven by the peak at 1.23 ppm [31]. The FTIR, ^1^H NMR and ^31^P NMR analyses have verified that the methylene phosphonic group successfully substituted the hydroxyl group, resulting from the reaction between κ-carrageenan, formaldehyde and phosphorous acid.

The degree of substitution of the methylene phosphonic group into κ-carrageenan was determined by the saponification method, using Eq 2. The calculated DS value of OMPC was 2.37.

### XRD analysis

**Fig 5:**
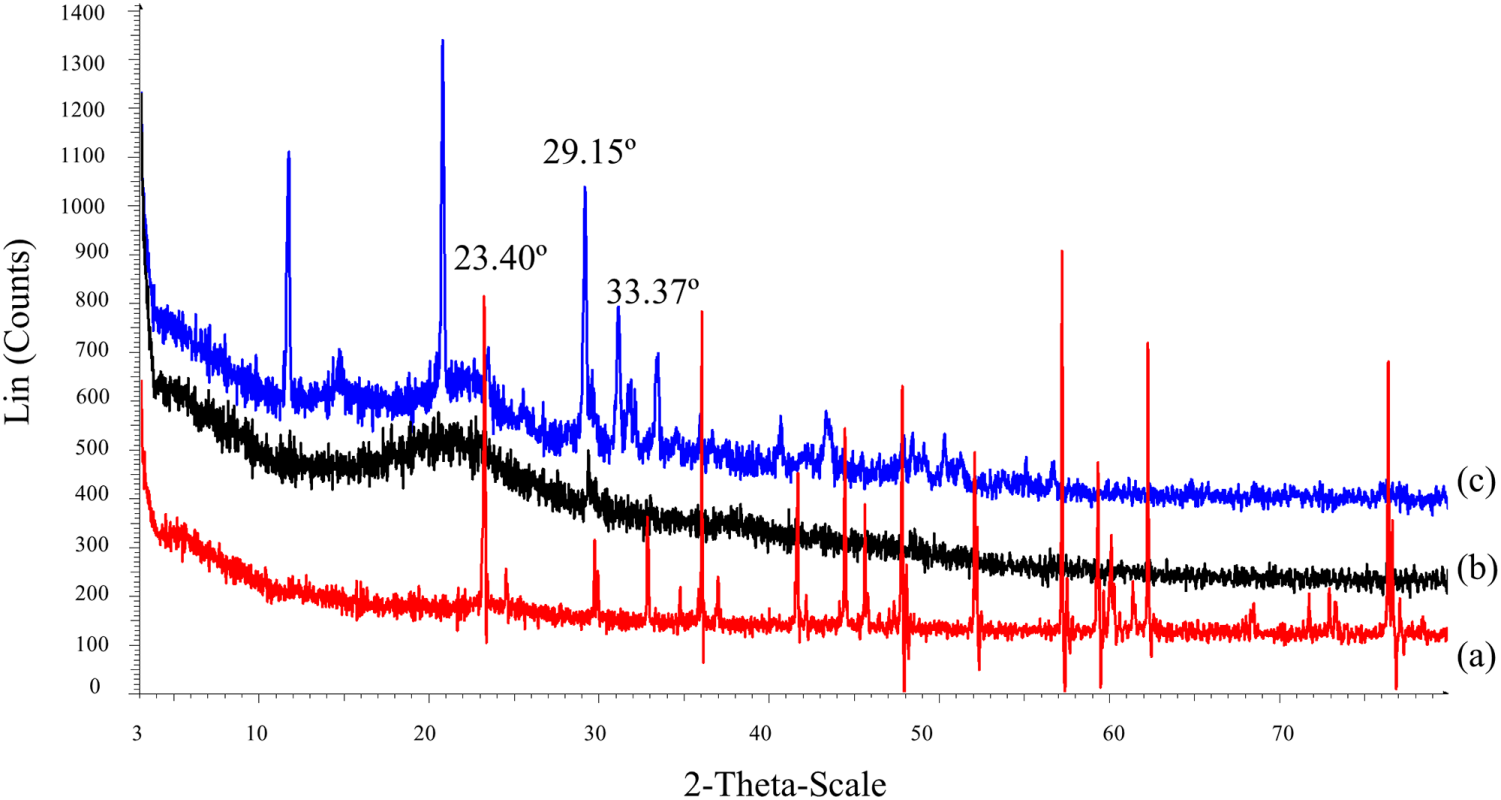
XRD diffractograms of (a) phosphorous acid, (b) κ-carrageenan and (c) OMPC.

X-ray diffractograms of κ-carrageenan, OMPC and phosphorous acid are shown in Fig 5. The XRD pattern of κ-carrageenan with a broad hump in the range of 10° to 25° reveals its semicrystalline nature. Compared with the XRD pattern of κ-carrageenan, the pattern of OMPC shows a combination of a broad peak contributed by semicrystalline κ-carrageenan and some distinct peaks contributed by crystalline phosphorous acid and unknown phases. From these sharp peaks, it can be concluded that OMPC possess a higher crystallinity than of κ-carrageenan. The introduction of methylene phosphonic groups has reoriented the inter- and/or intramolecular structure of κ-carrageenan to form more ordered regions. The increased crystallinity of OMPC compared with that of κ-carrageenan is consistent with the morphology of the OMPC film, which has a rougher surface.

### Thermal analysis

**Fig 6:**
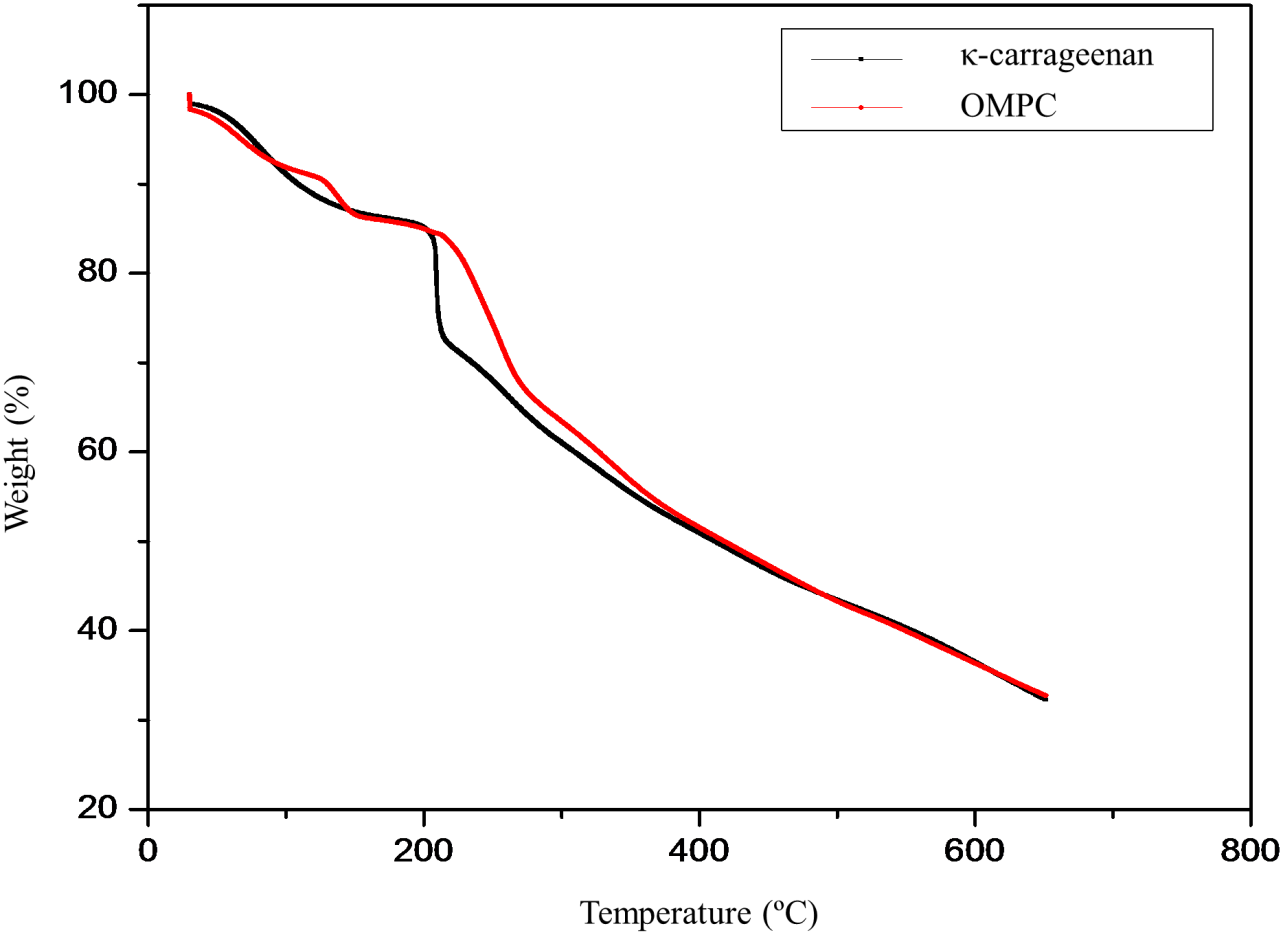
TG curves of κ-carrageenan and OMPC.

The thermograms of κ-carrageenan and modified κ-carrageenan are shown in the Fig 6. There are two stages of degradation in the κ-carrageenan sample. The first degradation stage was between 30°C and 100°C, showing approximately 11.3% of weight loss. The weight loss in the first stage is most likely due to the loss of moisture in the polymer, as polysaccharides usually have a strong affinity for water and are easily hydrated [22, 32, 33]. The second degradation stage started at 150°C with a drastic weight loss of 17.4% at a T_max_ of 209.34°C. This weight loss is associated with the degradation of sulphur dioxide from the polymeric backbone [32, 34].

On the other hand, there were three degradation stages observed for the OMPC sample. Similar to κ-carrageenan, the initial weight loss occurring below 100°C is due to the evaporation of water and solvent trapped in the polymer. With a further increased in temperature, there was a steep weight loss between 120°C to 170°C, which can be attributed to the loss of phosphonic acid units [35]. As the temperature increased, a significant weight loss of 21.26% was observed over the temperature range of 210°C to 280°C. The weight loss is attributed to the decomposition of –OSO_3_- group from the pendant chains attached to the polymeric backbone. Above 280°C, the degradation is caused by the fragmentation of carbohydrate backbone [34]. As shown by the less steep slope and higher degradation temperature of OMPC, the thermal stability of OMPC is slightly improved over that of κ-carrageenan because of its higher crystallinity content as confirmed by the XRD results [36]. The thermal analysis results also showed that both κ-carrageenan and modified κ-carrageenan were thermally stable up to 200°C, hence both polymers are suitable for electrochemical devices applications.

**Fig 7:**
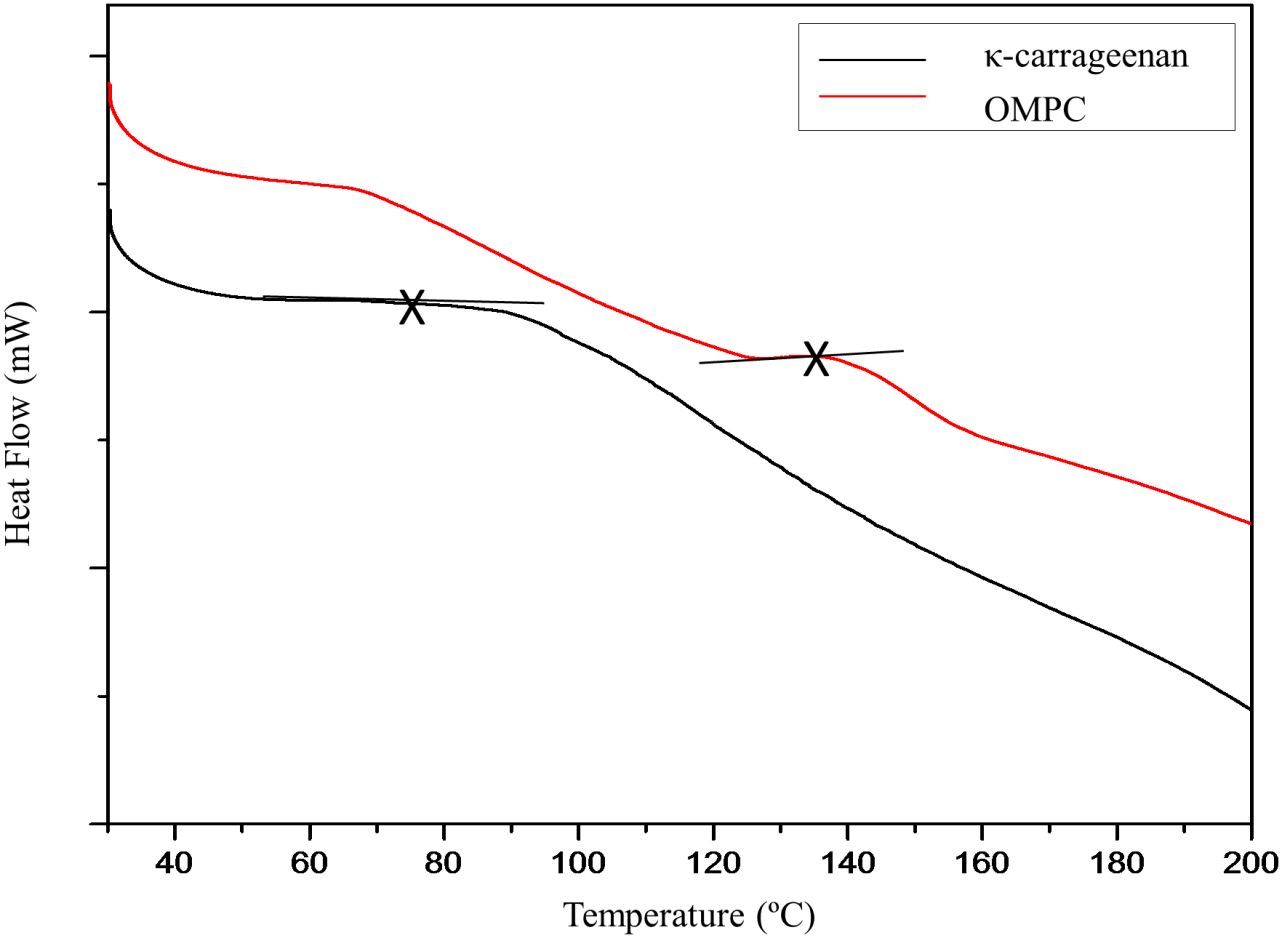
DSC thermograms of κ-carrageenan and OMPC.

Complementary to the TG analysis, DSC thermograms (Fig 7) were used to measure the glass transition temperature, T_g_, of the polymers. The slight endothermic curvature of OMPC near 65°C was attributed to absorbed water which as observed from the TG analysis. The DSC curves shows that OMPC has a higher T_g_ than κ-carrageenan. The T_g_ of OMPC is 137.36°C, whereas the T_g_ of κ-carrageenan is 73.02°C. The T_g_ of OMPC is higher than that of κ-carrageenan because the methylene groups enhanced the intra- and/or intermolecular interactions. Therefore, the segmental motion of the polymer chains was restricted, and hence, more energy to break the bonds [12]. In sum, the higher the crystallinity of a polymer, the higher its T_g_. Modified κ-carrageenan is more thermally stable than κ-carrageenan.

### VPSEM-EDX analysis

**Fig 8:**
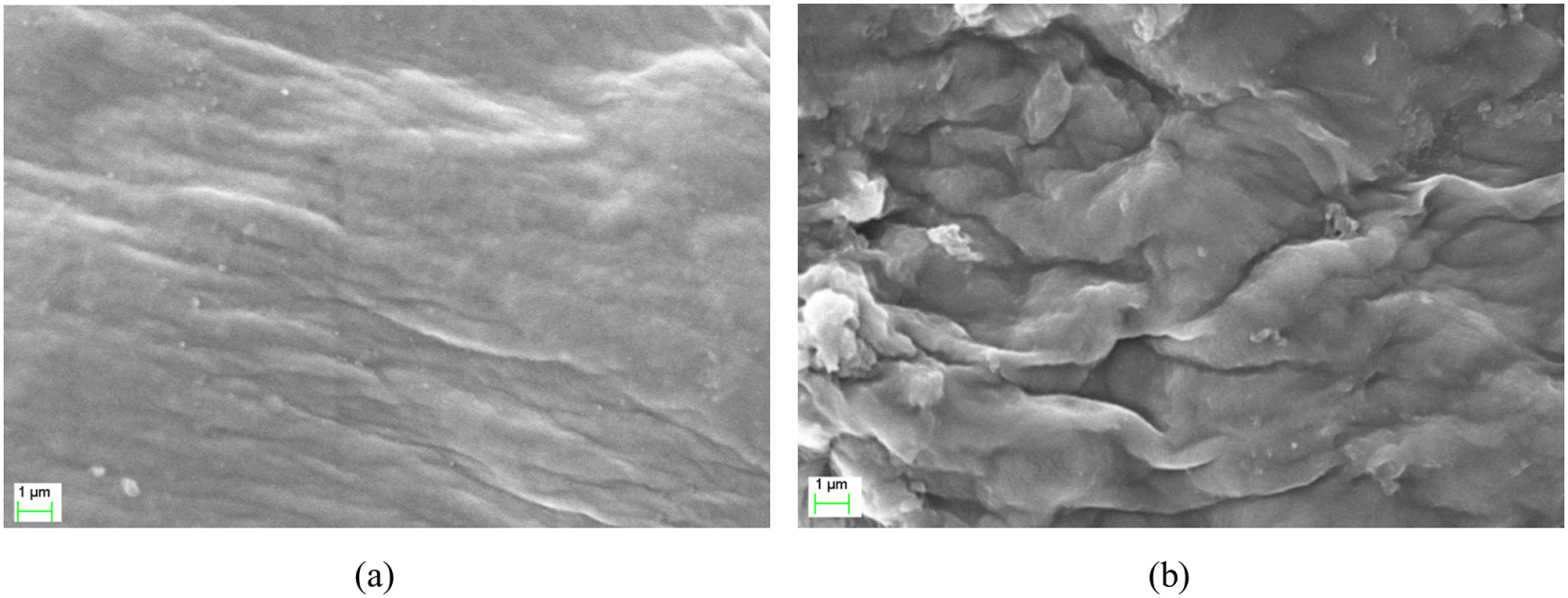
SEM micrographs for surfaces of (a) κ-carrageenan and (b) OMPC films.

**Fig 9:**
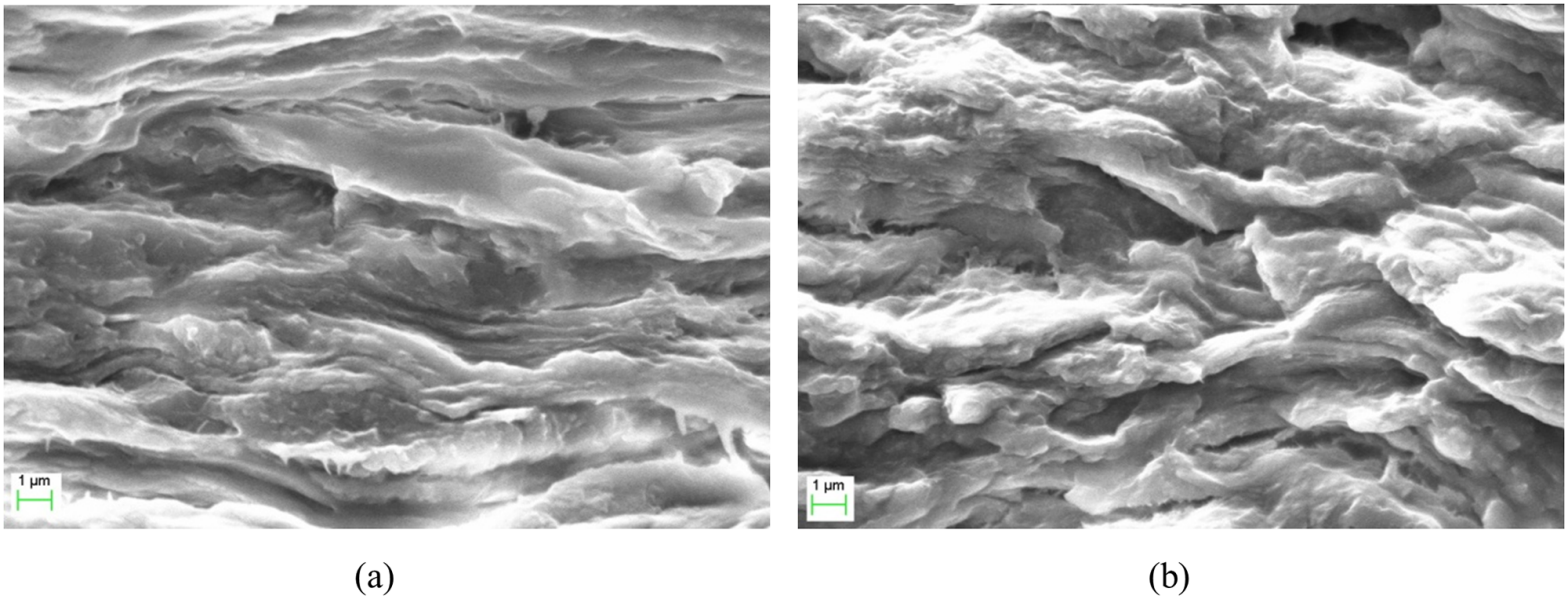
SEM micrographs for cross sections of (a) κ-carrageenan and (b) OMPC films.

The film morphology is an essential property of a polymer electrolyte [37]. The surface morphologies of the κ-carrageenan and OMPC films are shown in Fig 8. The surface morphology of the κ-carrageenan film was smooth and has a featureless topology compared with that of OMPC. The surface morphology of OMPC was uneven and rough, possibly due to the stronger intermolecular forces, resulting in higher surface tensions. Interestingly, the cross-sectional morphology of the films were not very difference. The cross-sectional morphology of the κ-carrageenan and OMPC films (Fig 9) present a homogeneous surfaces and close-packed structure without phase separation. The surface and cross-sectional morphologies of the OMPC film were disordered and rough due to the presence of the crystalline phase [38]. In addition, EDX analysis, shown in Table 1, also confirmed the presence of phosphorus in modified κ-carrageenan.

**Table 1:** EDX analysis of κ-carrageenan and OMPC.

Element	Weight %		Atomic %	
	κ-carrageenan	OMPC	κ-carrageenan	OMPC
C K	41.55	38.60	55.07	50.03
O K	31.91	41.23	31.75	40.12
P K	-	4.17	-	2.1
S K	26.54	15.99	13.17	7.76

### Ionic conductivity

**Fig 10:**
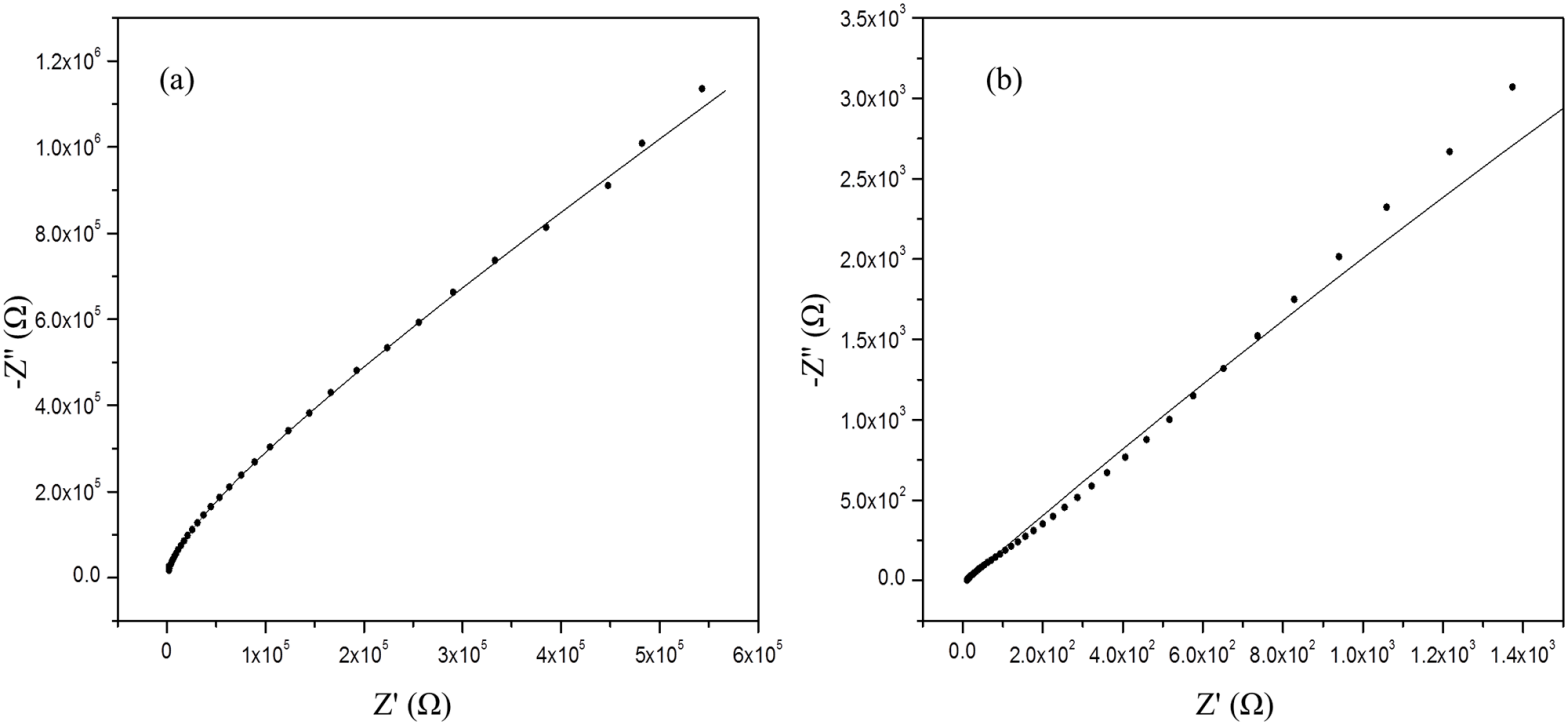
Impedance spectra of κ-carrageenan in (a) dry and (b) hydrated conditions.

**Fig 11:**
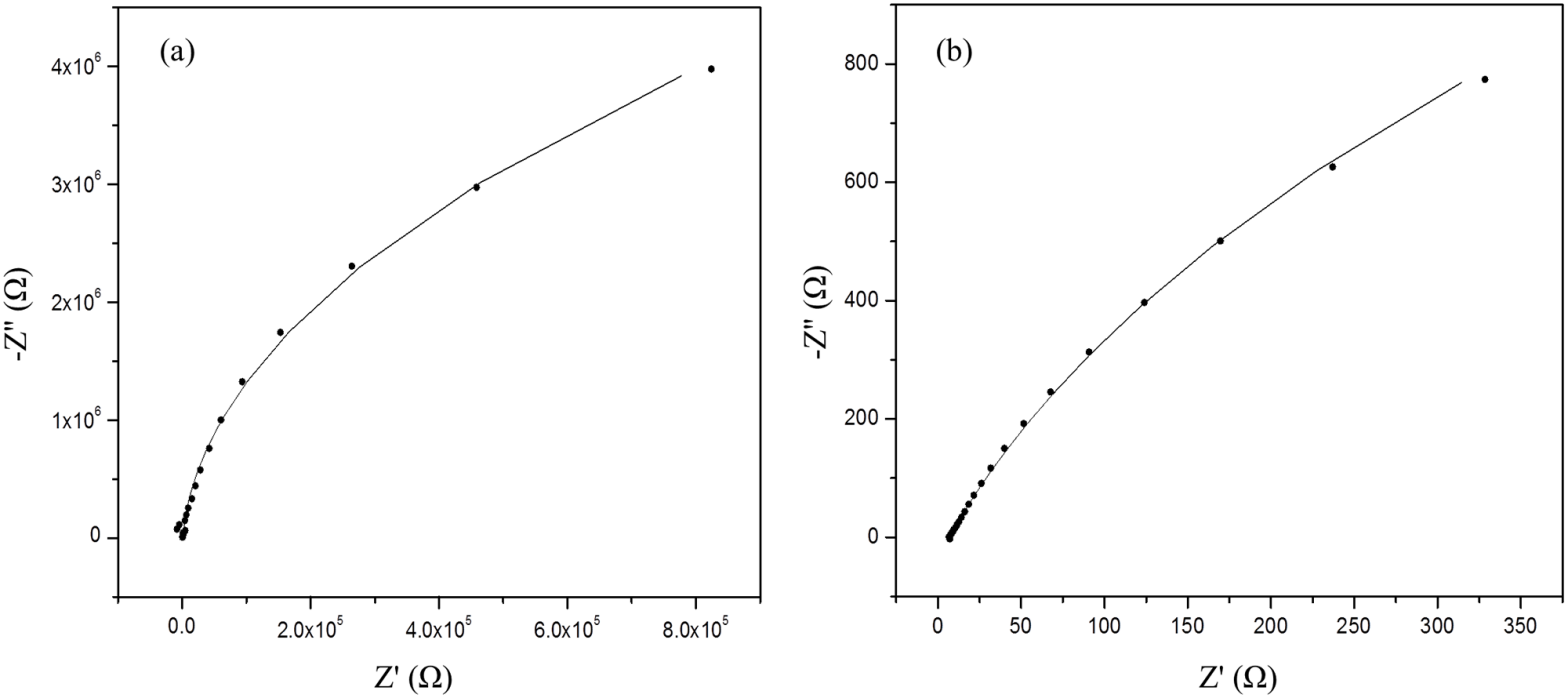
Impedance spectra of OMPC in (a) dry and (b) hydrated conditions.

Ionic conductivity measurements were carried out on both κ-carrageenan electrolytes before and after hydration. The Nyquist plots in Figs 10 and 11 show the impedance spectra of both κ-carrageenan and OMPC in the dried and hydrated state. The data (dots) in the Nyquist plots were fitted with an equivalent circuit (line) to estimate the bulk resistance of the polymer electrolyte. The ionic conductivity of the films was calculated using the formula given above.

Measurement of the dried membrane showed that the ionic conductivity of OMPC (1.54 × 10^−5^ S cm^-1^) was an order magnitude higher than that of κ-carrageenan (2.79 × 10^−6^ S cm^-1^). The ionic conductivity of κ-carrageenan is influenced by the number of oxygen atoms in the sulphonic groups (-SO_3_-) and hydroxyl groups (-OH). The oxygen atoms in the structure have a lone pair of electrons that enable the formation of coordination bonds with H^+^ which result in the formation of polymer complexes. The ionic conductivity of OMPC was remarkably improved because the oxygen-rich ionogenic group (-CH_2_PO_3_H_2_) substituted into the side chain of κ-carrageenan increased the total number of oxygen atoms [38]. Moreover, the phosphonic acid groups and sulphonic acid groups of OMPC act as proton donors and acceptors with the formation of protonic defects which promotes intermolecular proton transfer. The movement of protons along the polymer chain will enhance the ionic conductivity.

Mobarak et al. found that the ionic conductivity of dried samples of carboxymethyl κ-carrageenan was three orders of magnitude than that of untreated κ-carrageenan at room temperature [19]. They concluded that the improvement was affected by the morphology and the crystallinity of the membranes, where a smoother cross-section morphology improved the segmental motion in the polymer chains and enabled the conducting ions to move more freely in the electrolyte. In contrast to this work, phosphorous treated κ-carrageenan has a rougher cross-section morphology, higher glass transition temperature and larger crystalline phase, and these properties restrict the segmental motion of polymer chains [16]. Therefore, it is suggested that the improved ionic conductivity of OMPC is solely contributed by the increased concentration of oxygen active sites that promote proton migration.

The films easily swelled and disintegrated when immersed in water due to the hydrophilic properties of κ-carrageenan [39]. Therefore, the conductivity measurement was performed immediately after dipping the samples in water. Under humidified conditions, the water absorption ability is important for proton conductivity [12]. The κ-carrageenan polymer is a hydrophilic polyelectrolyte with good proton conductivity under humidified conditions. Table 2 shows that the ionic conductivity of both the hydrated films of κ-carrageenan and OMPC samples were enhanced by two orders of magnitude compared with dried films; and the ionic conductivity of hydrated OMPC is an order of magnitude higher than that of κ-carrageenan. The ionic conductivity of the κ-carrageenan membrane involves proton dissociation from the –SO_3_H groups and their transference to water molecules [11]. Accordingly, the substitution of –PO_3_H_2_ groups into κ-carrageenan increases the ionic conductivity of OMPC by the dissociation of protons from the phosphonic group, which increases the number of protons and promotes ion transfer through the membrane. The detachment of protons creates more free voids for ions to hop from one active site to the next. Furthermore, phosphonic acid is also known to have a great ability to retain water, providing additional proton-transfer pathways around hydrophilic regions [40]. Hence, the migration of protons in the hydrated membranes occurred through the hydrogen-bonded networks of absorbed water molecules.

**Table 2:** Ionic conductivity from EIS analysis.

Sample	Ionic conductivity (S cm^-1^)	
	before hydration	after hydration
κ-carrageenan	2.79 × 10^−6^	7.41 × 10^−4^
OMPC	1.54 × 10^−5^	2.26 × 10^−3^

In brief, the addition of methylene phosphonic groups into κ-carrageenan promoted the ionic conductivity by providing more active sites at which proton transfer can occur. For the hydrated membrane, the ionic conductivity was due to the hydrophilic nature of κ-carrageenan and the more hydrophilic region in the OMPC, which results from the –PO_3_H_2_ groups.

## Conclusions

An O-methylene phosphonic κ-carrageenan-based polymer electrolyte, OMPC, was successfully synthesized using formaldehyde and phosphorous acid. The structure of OMPC was confirmed via FTIR, ^1^H NMR, ^31^P NMR and SEM-EDX analyses. The polymer electrolyte membrane was produced by a solution casting method. The ionic conductivity of OMPC was improved over that κ-carrageenan, based on the results of impedance analysis. The OMPC shows potential as a polymer electrolyte membrane due to its considerably high ionic conductivity when hydrated. Further research should focus on improving the segmental motion of polymer chains in the membrane and strengthening the hydrated membrane.
